# The Mineralocorticoid Agonist Fludrocortisone Promotes Survival and Proliferation of Adult Hippocampal Progenitors

**DOI:** 10.3389/fendo.2016.00066

**Published:** 2016-06-16

**Authors:** Iacopo Gesmundo, Tania Villanova, Eleonora Gargantini, Emanuela Arvat, Ezio Ghigo, Riccarda Granata

**Affiliations:** ^1^Laboratory of Molecular and Cellular Endocrinology, Department of Medical Sciences, University of Torino, Torino, Italy; ^2^Department of Medical Sciences, Division of Endocrinology, Diabetes and Metabolism, University of Torino, Torino, Italy; ^3^Department of Medical Sciences, Division of Oncological Endocrinology, University of Torino, Torino, Italy

**Keywords:** mineralocorticoid receptor agonists, glucocorticoid receptor agonists, hippocampal progenitors, hippocampal progenitor cell proliferation and survival, amyloid beta peptide

## Abstract

Glucocorticoid receptor (GR) activation has been shown to reduce adult hippocampal progenitor cell proliferation and neurogenesis. By contrast, mineralocorticoid receptor (MR) signaling is associated with neuronal survival in the dentate gyrus of the hippocampus, and impairment of hippocampal MR has been linked to pathological conditions, such as depression or neurodegenerative disorders. Here, we aimed to further clarify the protective role of MR in adult hippocampal neurons by studying the survival and proliferative effects of the highly potent MR agonist fludrocortisone (Fludro) in adult rat hippocampal progenitor cells (AHPs), along with the associated signaling mechanisms. Fludro, which upregulated MR but not GR expression, increased survival and proliferation and prevented apoptosis in AHPs cultured in growth factor-deprived medium. These effects were blunted by the MR antagonist spironolactone and by high doses of the GR agonist dexamethasone. Moreover, they involved signaling through cAMP/protein kinase A (PKA)/cAMP response element-binding protein, phosphoinositide 3-kinase (PI3K)/Akt and its downstream targets glycogen synthase kinase-3β (GSK-3β) and mammalian target of rapamycin. Furthermore, Fludro attenuated the detrimental effects of amyloid-β peptide 1–42 (Aβ_1–42_) on cell survival, proliferation, and apoptosis in AHPs, and increased the phosphorylation of both PI3K/Akt and GSK-3β, which was reduced by Aβ_1–42_. Finally, Fludro blocked Aβ_1–42_-induced hyperphosphorylation of Tau protein, which is a main feature of Alzheimer’s disease. Overall, these results are the first to show the protective and proliferative role of Fludro in AHPs, suggesting the potential therapeutic importance of targeting MR for increasing hippocampal neurogenesis and for treating neurodegenerative diseases.

## Introduction

The hypothalamic–pituitary–adrenal (HPA) axis is the endocrine center of the stress system, consisting of corticotrophin-releasing hormone (CRH), pituitary adrenocorticotropic hormone (ACTH), and adrenal corticosteroid release. At the central level, corticosteroids influence neuronal plasticity and excitability, neurogenesis and neuronal death, as well as neuroendocrine control and behavioral responses (1). Their actions in the brain are mediated by glucocorticoid receptors (GRs) and mineralocorticoid receptors (MRs), which act in the cell nuclei as activators of gene transcription factors. MR expression is particularly high in the hippocampus and other limbic regions, where it co-localizes with GRs, which bind corticosteroid with a 10-fold lower affinity (3). GRs, in turn, are expressed throughout the brain, in neurons and glial cells, and are most abundant in hypothalamic neurons and corticotroph cells (2). Due to the differences in affinity, MR is always occupied by corticosterone, even at basal levels of HPA axis activity; however, higher hormone levels, after stress or circadian peak, progressively saturate the GR (3). Recently, rapid non-genomic effects have been demonstrated for MR and GR, which act as low-affinity membrane-associated receptor variants in response to rising corticosteroid levels (4).

Adult neurogenesis, which mainly occurs in the hippocampal subgranular zone of the dentate gyrus, is a tightly coordinated process involving proliferation, migration, and differentiation of progenitor cells. Numerous signal transduction mechanisms have been shown involved in hippocampal synaptic plasticity, memory formation, and neuronal cell proliferation and survival. These include cAMP/protein kinase A (PKA) and cAMP response element-binding protein (CREB) signaling, which play a central role in different steps of adult neurogenesis (5–7), and the phosphatidylinositol 3-kinase (PI3K)/Akt pathway, a key regulator of hippocampal progenitor cell proliferation and survival, also required for the mitogenic effects of several growth factors (8–10). Interestingly, PI3K/Akt was found to promote CREB phosphorylation, suggesting cross-talk mechanisms between these pathways (9). In addition, the downstream effectors of PI3K/Akt, glycogen synthase kinase-3β (GSK-3β) and mammalian target of rapamycin (mTOR) have been found involved in hippocampal synaptic plasticity and neuronal regeneration, and their signaling has been shown to be impaired in neurodegenerative disorders, including Alzheimer’s disease (AD) (11, 12).

Adult neurogenesis is also regulated by a variety of factors, including environmental factors, stress, hormones, and drugs (13, 14). Among the different hormones, elevation of glucocorticoids or administration of exogenous glucocorticoids has been shown to decrease progenitor cell proliferation and hippocampal neurogenesis (15–21), and adrenalectomy-induced depletion of glucocorticoids was found to attenuate these detrimental effects (22). On the other hand, several studies have shown that, differently from GR, MR activation is involved in hippocampal neuronal survival (4, 23). Indeed, deletion of MR in mice was found to reduce granule cell neurogenesis (24) and to impair learning ability (25), whereas MR overexpression increased differentiation and survival of embryonic stem (ES) cell-derived neurons (26); furthermore, forebrain MR overexpression enhanced memory, reduced anxiety, and attenuated neuronal loss in mouse cerebral ischemia (27). In addition, the natural MR agonist aldosterone was shown to counteract apoptotic hippocampal cell death induced by the GR agonist dexamethasone (DEX) (28) and to enhance neurogenesis in adrenalectomized rats (29). In humans, reduced MR expression and/or MR antagonism were found to be detrimental for the central functions and to affect HPA axis activity, suggesting that hippocampal MR, more than GR, might have clinical relevance in pathological conditions, such as depression, anxiety, or neurodegenerative diseases (30, 31).

Interestingly, different studies have shown impairment of the HPA axis and high glucocorticoid levels in animal models of AD (32, 33). Indeed, AD is an irreversible, progressive age-related neurodegenerative disorder that slowly destroys memory and thinking skills, and the hippocampus is one of the first brain regions to be damaged. Furthermore, it is characterized by accumulation of insoluble extraneuronal senile plaques, mainly composed of amyloid-β peptide (Aβ) and intraneuronal deposits of neurofibrillary tangles (NFTs), formed by hyperphosphorylated Tau protein, which cause massive neuronal death (34, 35).

Although a role for glucocorticoids and GR has been suggested in the progression of AD (33), to date, the direct role of MR agonists on Aβ-induced toxicity has not been investigated and, based on the survival effects in neurons, it can be hypothesized an MR-induced protection against Aβ peptide-induced detrimental effects in hippocampal cells.

The synthetic potent and selective MR agonist fludrocortisone (Fludro) has been previously found to display beneficial effects on executive function and memory in young depressed patients, as well as younger and older healthy individuals (36, 37); however, its role on neuronal cell survival has never been assessed. Therefore, based on the foregoing, the present study aimed to determine whether Fludro exerts survival and proliferative actions in adult rat hippocampal progenitor cells (AHPs), in stress conditions, such as growth factor deprivation, and Aβ-induced toxicity. In addition to the MR agonist, the role of the MR selective antagonist spironolactone (Spiro) and of the GR agonist DEX was also investigated, together with the underlying signaling pathways.

## Materials and Methods

### Reagents

Fludrocortisone, Spiro, DEX, β-amyloid fragment (1–42), wortmannin, rapamycin, 3-[4,5-dimethylthiazol-2-yl]-2,5-diphenyl tetrazolium bromide (MTT), Stemline neural stem cell medium, polyornithine, bovine serum albumin (BSA), and primers for RT-PCR were from Sigma-Aldrich (Milano, Italy). Human b-FGF, penicillin, streptomycin, fungizone, and trypsin were from Life Technologies, Inc. (Invitrogen, Milano, Italy). Quantikine immunoassay caspase-3 Colorimetric kit was provided by Assay Designs, Bologna, Italy. KT5720 was from Biomol Research Laboratory Inc. (DBA, Italy). Rabbit polyclonal antibodies P-Akt (Ser473), P-GSK-3β (Ser9), P-CREB (Ser133), and P-p70S6K (Thr389); mouse monoclonal antibody for P-Tau (Ser396) (PHF13) was from Cell Signalling Technology (Euroclone SpA, Milano, Italy). Total antibodies were from Abcam (Cambridge, UK). RT-PCR and Real-Time PCR reagents were from Life Technologies, Inc. (Invitrogen, Milano, Italy). Primers for RT-PCR were from TibMolBiol (Genova, Italy).

### Cell Culture and Treatments

The clonal population of AHP cells was a kind gift from Prof. Jorgen Isgaard (Laboratory of Experimental Endocrinology, Sahlgrenska Academy, University of Gothenburg, Sweden). The cells were isolated and cultured as previously described (10, 38). Briefly, AHPs were grown in polyornithine poly-coated flasks or wells. For normal proliferating conditions, they were cultured in Stemline neural stem cell medium, supplemented with 20 ng/ml of human b-FGF [normal medium (NM)] at 37°C in a 5% CO_2_ humidified atmosphere. For experimental conditions, the cells were switched to Dulbecco’s Modified Eagle’s medium (DMEM)/F12 (Sigma-Aldrich, Milano, Italy), supplemented with 0.1% BSA without growth factors and b-FGF for 12 h (control medium, c), then replaced with the same fresh medium, in either absence or presence of the different stimuli for further 24 h. For Aβ_1–42_ treatments, AHPs were maintained in control medium for 24 h and after removal of the medium they were incubated for the indicated times with Aβ_1–42_ and either with or without Fludro.

### Cell Survival and Proliferation

Cells were seeded in NM in polyornithine-coated 96-well plates at a cell concentration of 5 × 10^3^ cells/well. After 48 h, the medium was changed to control medium and cells were treated with different stimuli for further 24 h. Cell survival was assessed by MTT assay. The cells were incubated with 1 mg/ml MTT for approximately 1 h, then the medium was aspirated and the formazan product solubilized with 100 μl dimethyl sulfoxide (DMSO). Cell viability was assessed by spectrophotometry at 570 nm absorbance using the LT-4000 microplate reader (Euroclone, Milano, Italy). Cell proliferation was assessed using the 5-bromo-2-deoxyuridine (BrdU) incorporation ELISA (Roche Diagnostic SpA, Milano, Italy). Briefly, cells were incubated with BrdU labeling solution for 2 h at 37°C. After removal of the labeling solution, they were fixed, denatured, and incubated for 90 min with anti-BrdU antibody conjugate, which was subsequently removed by rinsing three times. Finally, the cells were incubated in substrate solution at room temperature and proliferation assessed by colorimetric detection at 450 nm absorbance using the LT-4000 microplate reader (Euroclone, Milano, Italy).

### Caspase-3 Activity

Caspase-3 activity was assessed by Caspase-3 Colorimetric kit (Assay Designs, Bologna, Italy) in cell lysates, according to the manufacturer’s instruction. Caspase-3 activity was assessed by colorimetric detection at 450 nm absorbance using the LT-4000 microplate reader (Euroclone, Milano, Italy).

### cAMP Assay

Cells were seeded in NM in polyornithine-coated 6-well dishes at a concentration of 2 × 10^5^ cells. After 48 h, the medium was changed to control medium and the cells were incubated in the presence of 100 μM of 3-isobutyl-1-methylxanthine (IBMX) and with or without Fludro 1 μM at the indicated time. cAMP was measured from cell lysates using the Cyclic AMP Assay (R&D System, Space Srl, Milano, Italy), as previously described (10). Briefly after incubations, the medium was removed and the cells lysed with ice-cold 0.1 N HCl. Cell lysates were centrifuged for 10 min at 800 rpm, and cAMP in the cell lysates was measured according to the manufacturer’s instructions.

### RT-PCR and Real-Time PCR

Total RNA extraction from AHP cells and reverse transcription to cDNA from 3 μg RNA were performed as previously described (39). Nine microliters of cDNA were amplified by standard polymerase chain reaction (PCR) in a 50 μl volume using AmpliTaq Gold Polymerase in a GeneAmp PCR System (Perkin Elmer, Milano, Italy) as described (10). The following primer pairs were used for both RT-PCR and real time PCR: MR (40), forward 5′-TACGACAATTCCAAGCCCGACACC-3′, reverse 5′-TACCTTGGCCCACTTCACGACCTG-3′ (99 bp) (NM_013131). GR (40), forward 5′-AGGGGAGGGGGAGCGTAATGG-3, reverse 5′-CCTCTGCTGCTTGGAATCTGC-3′ (119 bp) (AY293740); rat 18S rRNA, forward 5′-GTGGAGCGATTTGTCTGGTT-3′, and reverse 5′-CGCTGAGCCAGTTCAGTGTA-3′(X01117). Amplification for 18S rRNA subunit was used as internal control. For real-time PCR, cDNA was treated with DNA-free DNAse (LifeTech, Monza, Italy). Real-time PCR was performed with 50 ng cDNA, 100 nmol/L of each primer and the IQ-SYBR-green mastermix (Bio-Rad, Milano, Italy) using the ABI-Prism 7300 (Applied Biosystems).

### Western Blot Analysis

Immunoblot analysis was performed as previously described (10, 39). Proteins (50 μg) were resolved in 11% SDS-PAGE and transferred to a nitrocellulose membrane; after blocking with 1% BSA in Tris-buffered saline with 0.1% Tween for 2 h at room temperature, membranes were incubated overnight at 4°C with the specific antibody (P-CREB, P-Akt, P-GSK-3β, P-p70S6K, and P-Tau) (dilution 1:1000). Blots were reprobed with the respective total antibodies for normalization. Immunoreactive proteins were visualized using horseradish peroxidase-conjugated goat anti-mouse or goat anti-rabbit (1:5000) by enhanced chemiluminescence using ChemiDoc XRS (Bio-Rad, Milano, Italy) and densitometric analysis performed with Quantity One software (Bio-Rad).

### Statistical Analysis

Data are expressed as means ± SE. Results were analyzed using two-tailed Student *t*-test or two-way ANOVA followed by Tukey honestly significant difference for post-ANOVA comparisons (GraphPad Prism 5.0 Software, San Diego, CA, USA). Significance was established when *P* < 0.05.

## Results

### MR and GR Expression in AHP Cells

**Figure 1:**
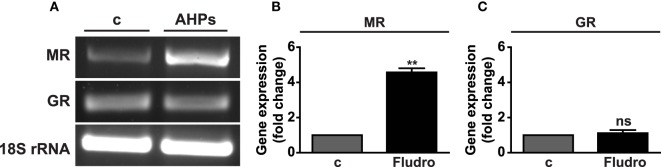
**MR and GR mRNA expression in AHPs**. **(A)** Representative images of MR and GR mRNA assessed by RT-PCR. Rat brain was used as positive control (c); 18S rRNA served as internal control. MR **(B)** and GR **(C)** gene expression assessed by real-time PCR in AHPs cultured in growth factor-deprived medium (c, control), in either absence or presence of Fludro (1 μM) for 24 h. mRNA expression was normalized to 18S rRNA and reported as fold increase vs. control. Results are the mean ± SEM of at least three independent experiments, each performed in duplicate. **P < 0.01 vs. c; ns, not significant.

MR and GR mRNAs were found expressed in AHPs, as assessed by RT-PCR (Figure 1A). Moreover, real-time PCR analysis showed that treatment with Fludro (1 μM) for 24 h strongly increased MR gene expression in AHPs (Figure 1B), whereas no variation was observed for GR (Figure 1C).

### Fludro Promotes Survival and Proliferation of AHPs and Prevents Apoptosis

**Figure 2:**
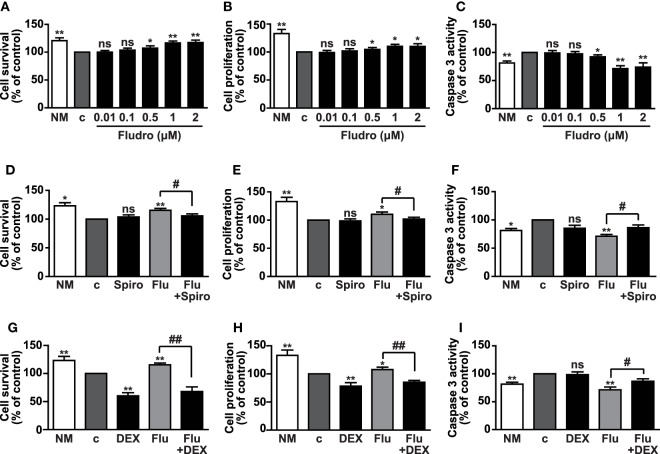
**Fludro survival, proliferative, and antiapoptotic effects in AHPs**. The cells were cultured in either normal medium (NM) or in growth factor-deprived medium (c, control medium) for 12 h and then for further 24 h with Fludro alone, at the indicated concentrations **(A–C)**, or with 1 μM Fludro (Flu) with or without either 1 μM Spiro **(D–F)** or 5 μM DEX **(G–I)**. Cell survival and proliferation were assessed by MTT and BrdU incorporation assays, respectively, and apoptosis by caspase-3 activity. Results, expressed as percentage of control, are the mean ± SE of three independent experiments. *P < 0.05, **P < 0.01 vs. c; ^#^*P* < 0.05, ^##^*P* < 0.01; ns, not significant.

Once ascertained MR and GR expression, we sought to determine the biological effects induced by different concentrations (0.01–2 μM) of the potent synthetic MR agonist Fludro in AHPs, which were cultured in growth factor-deprived medium (referred to as control medium). As previously reported (10, 38), cell survival and proliferation were reduced in control, with respect to growth factor-containing medium (NM) (Figures 2A,B). However, cell survival and proliferation progressively increased in AHPs treated with Fludro, with respect to NM. Fludro displayed these effects at the highest concentrations tested, namely from 0.5 μM and particularly, at 1 and 2 μM (Figures 2A,B). We next determined the potential antiapoptotic role of the MR agonist and observed that, at the same concentrations, Fludro strongly reduced serum starvation-induced apoptosis to levels that were even below those of control, as assessed by reduction of caspase-3 activity (Figure 2C). Therefore, following these results, 1 μM was selected as the best Fludro concentration for the subsequent experiments.

To next determine the involvement of MR in the effects of Fludro, AHPs were treated with Fludro, either alone or in combination with the MR selective antagonist Spiro (41). Fludro survival, proliferative, and antiapoptotic actions were found reduced by cotreatment with Spiro (Figures 2D–F). Interestingly, the protective effects of Fludro were also abolished by high concentrations (5 μM) (4) of the GR agonist DEX (Figures 2G–I), which was previously shown to inhibit the proliferation of hippocampal progenitors (17, 42).

Taken together, these results suggest that Fludro elicits protective effects in AHPs, which are counteracted by either selective MR antagonists or high concentrations of GR agonists.

### Fludro Survival and Proliferative Effects Involve Signaling Through cAMP/Protein PKA and CREB

**Figure 3:**
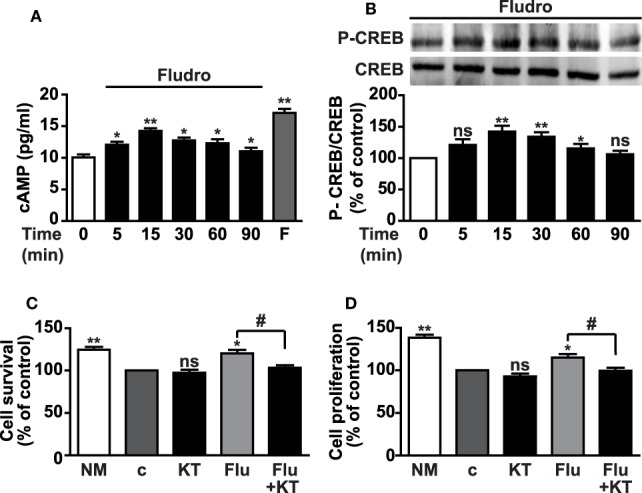
**Involvement of cAMP/PKA/CREB signaling in Fludro survival and proliferative effects**. **(A)** Intracellular cAMP levels in growth factor-deprived AHPs cultured for the indicated times with 1 μM Fludro, in the presence of phosphodiesterase inhibitor IBMX (100 μM), that was added 30 min before stimulation. Forskolin (F) (50 μM for 2 min) was used as positive control. Results are the mean ± SE of three independent experiments (*P < 0.05, **P < 0.01 vs. time 0). **(B)** CREB phosphorylation on serine 133 (top panel) evaluated by Western blot in lysates from AHPs incubated with Fludro (1 μM) for the indicated times. Equal protein loading was determined by reprobing with total CREB antibody (bottom panel). Blots are representative of three independent experiments. Graphs show the densitometric analysis of phosphorylated proteins normalized to total proteins and reported as percentage of basal (*P < 0.05 and **P < 0.01 vs. time 0; ns, not significant). **(C,D)** Cell survival and proliferation assessed by MTT assay and BrdU, respectively, in AHP cells cultured in normal medium (NM) or in growth factor-deprived medium (c, control medium) for 12 h, and then for further 24 h with 0.5 μM KT5720 (KT), either alone or with 1 μM Fludro (Flu). Data, expressed as percentage of control, are the mean ± SE (*n* = 3); *P < 0.05, **P < 0.01 vs. c; ^#^*P* < 0.05; ns, not significant.

Activation of the cAMP/PKA/CREB cascade plays an important role in the regulation of adult neurogenesis (6, 7). Furthermore, elevation of cAMP levels and activation of CREB by the MR agonist aldosterone has been previously demonstrated in porcine coronary artery vascular smooth muscle cells through non-genomic effects (43), as well as the crosstalk between cAMP and aldosterone signaling in human hepatoma cell lines (44). Therefore, we next investigated whether Fludro-induced survival and proliferation would imply activation of the cAMP/PKA/CREB pathway. cAMP levels were found elevated by Fludro (1 μM), peaking at 15 min and being still significantly increased at 90 min with respect to untreated cells. The adenylyl cyclase activator forskolin was used as positive control and strongly increased cAMP levels, as expected (Figure 3A). Fludro also promoted the phosphorylation of CREB on serine 133, which peaked at 15 min and progressively returned to basal levels at 90 min (Figure 3B). The specific PKA inhibitor KT5720 abolished Fludro-induced survival and proliferation in cells cultured in growth factor-deprived medium, further suggesting the involvement of PKA/CREB signaling in the effects of the MR agonist (Figures 3C,D).

### Phosphorylation of PI3K/Akt, GSK-3β, and mTOR Is Required for Fludro Survival, Proliferative, and Antiapoptotic Effects

**Figure 4:**
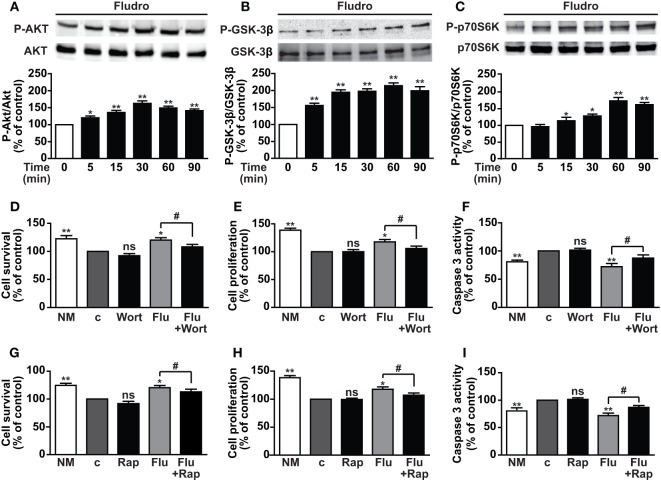
**Involvement of PI3K/Akt, GSK-3β, and mTOR/p70S6K signaling in Fludro effects**. **(A)** Akt, **(B)** GSK-3β, and **(C)** p70S6K phosphorylation evaluated by Western blot in AHPs treated with 1 μM Fludro for the indicated times (upper panels). Blots, each representative of three independent experiments, were reprobed with total antibodies for normalization (lower panels). Graphs show phosphorylated proteins normalized to total proteins and reported as percentage of basal (*P < 0.05 and **P < 0.01 vs. time 0). **(D–I)** Cell survival, proliferation, and apoptosis assessed by MTT, BrdU, and caspase-3 activity, respectively, in AHPs cultured in normal medium (NM) or in growth factor-deprived medium (c, control medium) for 12 h, and then for further 24 h with either wortmannin (Wort, 100 nM) **(D–F)** or rapamycin (Rap, 50 nM) **(G–I)**, both alone or with 1 μM Fludro (Flu). Data, expressed as percentage of c, are the mean ± SE of four independent experiments. *P < 0.05, **P < 0.01 vs. *c*; ^#^*P* < 0.05; ns, not significant.

Activation of the PI3K/Akt pathway has been shown to play a pivotal role in both survival and proliferation of adult hippocampal progenitors, also induced by different growth factors (5, 9, 10). Furthermore, PI3K/Akt-mediated inactivation of GSK-3β through phosphorylation on Ser9 residue is crucial for hippocampal neurogenesis and neuronal survival (11). Here, Fludro-treated AHPs showed a time-dependent increase of Akt phosphorylation, which peaked at 30 min and decreased thereafter, although being still significantly elevated at 60 and 90 min compared to basal (Figure 4A). Similarly, Fludro strongly and time-dependently increased GSK-3β phosphorylation at Ser9, which was maintained up to 90 min after stimulation (Figure 4B). The role of Fludro was also assessed on activation of the other Akt effector mTOR, (12). Specifically, we studied the effect of Fludro on the mTOR target p70S6K and found a time-dependent increase of its phosphorylation at Thr389, compared to basal (Figure 4C). Next, to further assess the role of Akt and mTOR in Fludro effects, AHPs were treated with the specific inhibitors of these pathways. Fludro survival, proliferative, and antiapoptotic actions were found reduced by either the Akt inhibitor wortmannin (Figures 4D–F) or the mTOR inhibitor rapamycin (Figures 4G–I), whereas these compounds alone were inactive. Overall, these results suggest that Fludro-induced protection in AHPs requires activation of PI3K/Akt and mTOR, as well as inactivation of GSK-3β.

### Fludro Counteracts the Detrimental Effects of Aβ Peptide 1–42

**Figure 5:**
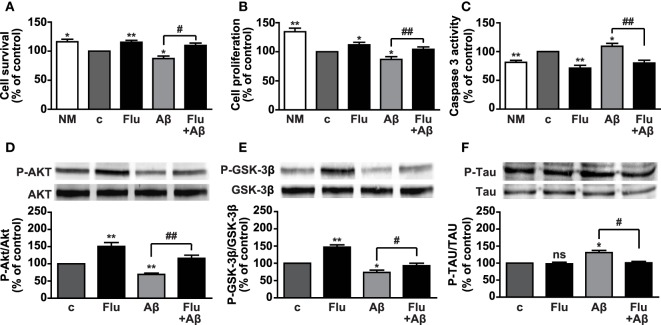
**Survival effects of Fludro against Aβ_1–42_-induced toxicity in AHPs**. **(A–C)** Cell survival, proliferation, and apoptosis in cells treated for 24 h in control medium (c) with or without Aβ_1–42_ (1 μM) and in either absence or presence of Fludro (1 μM). Data, expressed as percentage of c, are the mean ± SE of four replicates. *P < 0.05 and **P < 0.01 vs. c; ^#^*P* < 0.05 and ^##^*P* < 0.01; ns, not significant. **(D)** Akt, **(E)** GSK-3β, and **(F)** Tau phosphorylation (top panels) assessed by Western blot in cells treated with Aβ_1–42_ (1 μM) and Fludro (1 μM), either alone or in combination. The cells were pretreated with Fludro for 30 min and then treated with Aβ_1–42_ for 60 min. Blots were reprobed with total antibodies for normalization (lower panels). Graphs show phosphorylated proteins normalized to total proteins and reported as percentage of basal. *P < 0.05 and **P < 0.01 vs. c; #*P* < 0.05 and ^##^*P* < 0.01; ns, not significant (*n* = 3).

Aβ peptides exert toxic effects in a variety of neural cell types by reducing survival and proliferation. Indeed, abnormal Aβ peptide 1–42 (Aβ_1–42_) deposition is one of the main hallmarks of AD and contributes to neuronal apoptotic cell death in the hippocampus and cerebral cortex. Moreover, Aβ_1–42_ plays a critical role in the hyperphosphorylation of Tau protein and the consequent formation of NFTs (34, 35). Fludro-induced survival and proliferative effects observed in growth factor-deprived conditions led us to hypothesize a protective role of the MR agonist against Aβ_1–42_-induced cell death. In AHPs treated for 24 h with 1 μM Aβ_1–42_, whose concentration was selected on the basis of our previous findings (10), coincubation with Fludro attenuated the detrimental effect of the peptide by increasing survival and proliferation and restoring apoptosis to control levels (Figures 5A–C). Consistent with the previous results, Fludro, which *per se* promoted both Akt and GSK-3β phosphorylation, also counteracted Aβ_1–42_-induced reduction of Akt and GSK-3β phosphorylation (Figures 5D,E). Next, the effect of Fludro was examined on Tau protein, whose hyperphosphorylation by GSK-3β is involved in the formation of NFTs and the pathogenesis of AD (35, 45). Figure 5F shows that Fludro alone had no effect with respect to control; however, it completely blocked Aβ_1–42_-induced phosphorylation of Tau. Collectively, these findings suggest a protective role for Fludro against Aβ_1–42_-induced toxicity in hippocampal progenitors.

## Discussion

This study is the first to describe the survival, proliferative, and antiapoptotic effects of the MR agonist Fludro in adult rat hippocampal progenitors. Fludro counteracted the detrimental effects of growth factor deprivation through mechanisms involving activation of the cAMP/PKA/CREB signaling and PI3K/Akt and mTOR pathways, as well as inactivation of GSK-3β. Furthermore, Fludro reduced the negative effects of Aβ_1–42_ on AHP survival, proliferation, and apoptosis through Akt and GSK-3β-mediated signaling and inhibition of Aβ_1–42_-induced hyperphosphorylation of Tau protein.

Adult hippocampal neurogenesis comprises proliferation and differentiation of progenitors, survival and maturation of new neurons, and their integration into neuronal circuits. Defects in neurogenesis cause memory impairment and cognitive deficits and have been found to be critical events in aging and neurodegenerative diseases, including AD (13, 14). Both MR and GR are abundantly expressed in the limbic areas, where they display quite opposite effects (1, 2). Indeed, MRs play an essential role in the neuroendocrine and behavioral responses to stress and in cognitive functions, and are involved in the integrity and stability of neuronal networks (4, 23). On the other hand, activation of GR by high levels of glucocorticoids promotes neuronal death through cell cycle arrest and induction of apoptosis (17, 20, 21, 46). We show here expression of both MR and GR in AHPs and interestingly, MR, but not GR, mRNA expression was found increased by treatment with Fludro, suggesting a specific role for MR in mediating Fludro survival and proliferative effects. This assumption was also bolstered by the results showing that the MR antagonist Spiro was able to counteract the actions of Fludro in AHPs. Furthermore, previous studies have demonstrated that MR overexpression *in vitro* was associated with increased survival of rat primary cortical neurons, as well as neuroprotection in rat hippocampus and decreased sensitivity to stress (47). In addition, exposure to chronic stressors was previously found to downregulate hippocampal MR, leading to a reduced MR:GR ratio, and decreased hippocampal MRs have been associated with enhanced stress-induced HPA axis activity (48). Interestingly, antidepressant administration increased MR expression (49); furthermore mice with conditional forebrain-specific MR overexpression showed an attenuated HPA axis response to stress (50) and MR overexpression was recently shown to confer resilience to the effects of chronic stress on hippocampus-dependent function and structural plasticity (51). Moreover, murine ES cells that overexpressed human MR and were induced to differentiate into mature neurons showed increased neuron survival and increased ratio of anti- vs. proapoptotic molecules (26). Therefore, in our study increased MR expression may be a mechanism whereby Fludro promotes cell survival and counteracts apoptosis induced by growth factor deprivation.

Although the neuroprotective actions of MR and of natural MR agonists, such as aldosterone have been described (4, 23, 26–28), to the best of our knowledge, this is the first study showing the protective role of Fludro in hippocampal progenitors. We and others have recently reported that high doses of Fludro display inhibitory effects on the HPA axis in humans, likely through binding to hippocampal MR (52–54), whereas MR antagonism increased HPA axis activity (31). Accordingly, Fludro also enhanced efficacy of antidepressants and improved memory and executive functions in young depressed patients (48). These findings led us to hypothesize that the highly selective MR agonist Fludro, similarly to the natural agonist aldosterone, would display protective action in adult hippocampal progenitors. Indeed, we show here that Fludro counteracted the effects of growth factor deprivation in AHPs by promoting cell survival and proliferation, and reducing apoptosis. To date, very few studies have described the effects of Fludro on cells; among these, Fludro was found to stimulated cell viability in bladder cancer cell lines (55), whereas in neurons, it increased nerve growth factor (NGF)-induced neurite outgrowth in a neuronal model derived from PC12 pheochromocytoma line (56). Conversely, at variance with the previously observed survival actions of MR, a recent study demonstrated Fludro-induced neuron damage in pyramidal cells of the hippocampal CA3 region, which expressed only MR and not GR (57).

Herein, we show that the survival effects of Fludro in AHPs were attenuated not only by the MR antagonist Spiro, suggesting MR specificity, but also by the GR agonist DEX. In fact, as previously demonstrated in hippocampal cells (17), DEX alone strongly reduced cell survival and proliferation in growth factor-deprived AHPs, whereas it had no effect on apoptosis. Fludro was likely unable to counteract such a strong death response to GR activation; in addition, DEX also reduced the antiapoptotic effect of Fludro, although having no proapoptotic effect on its own. These results suggest that upon stress conditions, such as high DEX levels, MR response is unable to attenuate the detrimental effects of GR.

The limitation of the present study is that the experiments have been performed using a single cell type. However, the adult rat hippocampus-derived progenitors, employed herein, were originally isolated from the rat hippocampal dentate gyrus and are a well-established cell model, which have been thoroughly studied with regard to their proliferative capacities and lineage stability. Most importantly, they can generate the three main lineages of the central nervous system (8, 38, 58).

In addition to the classical long-lasting genomic mechanisms regulating gene transcription, MRs have been recently shown to influence cellular physiology through rapid non-genomic pathways (59). These are represented by low-affinity membrane receptors that are activated when corticosteroid levels rise, for example, shortly after stress exposure, and are most likely located on the presynaptic terminal in hippocampal neurons; moreover, their effects were found to be blocked by Spiro (4, 60). Furthermore, aldosterone has been shown to induce a rapid increase of cAMP levels and to promote CREB phosphorylation in vascular smooth muscle cells through non-genomic mechanisms; in this study, however, these effects were not blocked by the MR antagonist Spiro or by inhibitors of transcription and protein synthesis (43). Moreover, cAMP potentiated the activation of transcription by aldosterone of a GRE-containing promoter in different cell types (44). In line with these studies, we show here that Fludro rapidly increased cAMP in AHPs and also promoted the phosphorylation of CREB, suggesting activation of non-genomic pathways. Furthermore, Fludro-induced survival and proliferation was reduced by the PKA inhibitor KT5720, further supporting the implication of cAMP/PKA/CREB signaling, which is recognized as a central player in different steps of adult neurogenesis, including hippocampal cell survival, maturation, and integration of new neurons (6, 7).

Recently, rapid non-genomic effects of glucocorticoids were also observed in hippocampal neurons, where corticosterone promoted the activation of both PKA and PI3K/Akt (61); however, the role of MR on activation of survival signaling pathways is still quite unknown. Furthermore, the importance of PI3K/Akt activity on proliferation and survival of adult hippocampal progenitors (8, 9), as well as its association with CREB phosphorylation, has been previously described (9). Interestingly, we show here rapid Fludro-induced phosphorylation of Akt in AHPs; moreover, Akt inhibition by wortmannin reduced Fludro survival, proliferative, and antiapoptotic effects. Similarly, Fludro rapidly increased the phosphorylation of the PI3K/Akt downstream targets GSK-3β and mTOR/p70S6K, which resulted in their inactivation and activation, respectively. GSK-3β, a key component of the Wnt pathway, plays a crucial role in progenitor cell differentiation and survival during neurogenesis, as well as in neuronal survival (11). Furthermore, impaired GSK-3β activity has been associated with psychiatric disorders and neurodegenerative diseases, including AD (45). Interestingly, as for Akt, Fludro survival and mitogenic actions were reduced in the presence of the mTOR inhibitor rapamycin, suggesting rapamycin sensitive mTOR complex 1 (mTORC1)-mediated mechanisms. Accordingly, mTOR/p70S6K pathway has been linked to synaptic plasticity and neurogenesis, and mTOR signaling impairment has been related to neurodegenerative disorders, including autism and AD (12). Therefore, on the basis of the protective effects observed in growth factor-deprived conditions and the signaling pathways involved, we next sought to determine whether Fludro would protect AHPs from the detrimental effects of Aβ_1–42_ peptide. Indeed, Aβ_1–42_ is a main responsible for neuronal death, memory, and cognitive impairment in AD (34, 35). Furthermore, different studies have described the correlation between high glucocorticoid levels or dysregulation in the corticosteroid system and AD (32, 33). In fact, in both *in vitro* and *in vivo* models, stress-levels of glucocorticoids were found to increase Aβ formation and Tau accumulation, through GR-mediated mechanisms (62). Moreover, elevated glucocorticoid levels increased susceptibility of cholinergic neurons to Aβ_1–42_-mediated toxicity *in vivo* (63) and in hippocampal neuronal cultures (64). Conversely, CRH, which initiates neuroendocrine responses to stress by activating the HPA axis, was found to reduce cell death caused by an Aβ peptide in primary hippocampal and cortical neurons, an effect that was blocked by a CRH receptor antagonist and by an inhibitor of PKA (65). However, so far the direct effect of MR agonists on Aβ-induced toxicity in hippocampal cells has never been investigated. Here, we show for the first time that Fludro counteracts the detrimental effects of Aβ_1–42_ in AHPs, by increasing cell survival and proliferation and reducing apoptosis. Fludro also increased both Akt and GSK-3β phosphorylation, that was reduced by Aβ_1–42_, and restored Aβ_1–42_-induced phosphorylation of Tau to basal levels.

Overall, these findings are the first to show the protective and proliferative role of Fludro in adult hippocampal progenitors exposed to different stress stimuli, including those characteristics of neurodegenerative diseases. These effects likely involved both genomic and non-genomic mechanisms, as suggested by the activation of specific signaling pathways. Therefore, the results of this study further support the previously observed neuroprotective role of MR and suggest that MR agonists may represent a possible therapeutic strategy for reducing hippocampal cell loss, improving neurogenesis and for approaching neurodegenerative diseases, such as AD.

## Author Contributions

IG performed the experiments, analyzed the results, and prepared the paper draft; TV and E.Garg. contributed to the experiments and results’ analysis; EA and EG contributed to the conception of the work and critically revised the manuscript; RG designed the study, supervised the work and the results’ analysis, and wrote the paper.

## Conflict of Interest Statement

The authors declare that the research was conducted in the absence of any commercial or financial relationships that could be construed as a potential conflict of interest.

## Abbreviations

Aβ, amyloid-beta peptide; AD, Alzheimer’s disease; AHP, adult rat hippocampal progenitor cells; BrdU, 5-bromo-2-deoxyuridine; BSA, bovine serum albumin; cAMP, cyclic AMP; CREB, cAMP response element-binding protein; DEX, dexamethasone; DMEM, Dulbecco’s Modified Eagle’s medium; ELISA, enzyme-linked immunosorbent assay; ERK1/2, extracellular signal-regulated protein kinases 1 and 2; FGF, fibroblast growth factor; Fludro, fludrocortisone; GSK-3β, glycogen synthase kinase-3 beta; IBMX, 3-isobutyl-1-methylxanthine; mTOR, mammalian target of rapamycin; MTT, 3-(4,5-dimethylthiazol-2-yl)-2,5-diphenyltetrazolium bromide; NFTs, neurofibrillary tangles; NM, normal medium; p70S6K, p70 ribosomal S6 kinase; PI3K, phosphatidylinositol 3-kinase; PKA, protein kinase A; RT-PCR, reverse transcriptase-polymerase chain reaction; Spiro, spironolactone.
